# Molecular Electronic Angular Motion Transducer Broad Band Self-Noise

**DOI:** 10.3390/s151129378

**Published:** 2015-11-20

**Authors:** Dmitry Zaitsev, Vadim Agafonov, Egor Egorov, Alexander Antonov, Anna Shabalina

**Affiliations:** 1 Center for Molecular Electronics, Moscow Institute of Physics and Technology, Moscow 117303, Russia; E-Mails: agvadim@yandex.ru (V.A.); egorovev@mail.ru (E.E.); info@nordlab.com (A.A.); 2 NordLab LLC, Dolgoprudny, Moscow Region 141700, Russia; 3 R-sensors LLC, Dolgoprudny, Moscow Region 141700, Russia; E-Mail: btform@mail.ru

**Keywords:** molecular electronics, sensor, transducer, angular motion, noise, self-noise, rate, acceleration

## Abstract

Modern molecular electronic transfer (MET) angular motion sensors combine high technical characteristics with low cost. Self-noise is one of the key characteristics which determine applications for MET sensors. However, until the present there has not been a model describing the sensor noise in the complete operating frequency range. The present work reports the results of an experimental study of the self-noise level of such sensors in the frequency range of 0.01–200 Hz. Based on the experimental data, a theoretical model is developed. According to the model, self-noise is conditioned by thermal hydrodynamic fluctuations of the operating fluid flow in the frequency range of 0.01–2 Hz. At the frequency range of 2–100 Hz, the noise power spectral density has a specific inversely proportional dependence of the power spectral density on the frequency that could be attributed to convective processes. In the high frequency range of 100–200 Hz, the noise is conditioned by the voltage noise of the electronics module input stage operational amplifiers and is heavily reliant to the sensor electrical impedance. The presented results allow a deeper understanding of the molecular electronic sensor noise nature to suggest the ways to reduce it.

## 1. Introduction

There is a wide range of potential demand for compact, economical, low-noise sensors capable to measuring tiny vibrations of the objects to which the sensors are fixed. The main application areas are seismology and seismic exploration, where angular motion sensors based on a number of operating principles are being actively introduced. This has already resulted in a variety of new methods for multicomponent seismic observations where three components of ambience linear motion and three angular parts are registered simultaneously [1,2,3,4]. The molecular-electronic transfer (MET) technology is considered as one of the most prominent technologies for these areas. The advantage of modern molecular electronic angular motion sensors is their combination of high quality characteristics, especially a low level of self-noise, combined with low manufacturing cost, reliability and small size [5]. MET angular motion sensors could potentially be used in personal navigation systems, in the steering systems of space craft, submersible craft and robots, as well as in monitoring systems for complex engineering structures [6,7]. Besides, molecular electronic angular motion sensors represent an alternative to less accurate, less sensitive and noisier MEMS-based sensors in such application areas as home and in-car electronics, railway and air transport, unmanned aircraft systems, exo-skeletons, medical equipment, *etc.* [8,9,10]. One of the most significant technical parameters of all kinds of vibration sensors is their self-noise level. This value influences the accuracy of measurements, especially the ability to resolve tiny signals. A qualified comparison of this parameter for MET sensors with other types of angular vibration sensors is given in [11]. In accordance with [11], MET sensor self-noise is on the order of 10^−7^ rad/s/$\sqrt{\text{Hz}}$ rms in the bandwidth near 1 Hz, which is 100 times lower than the same values in modern MEMS and Fiber Optics Gyros (FOGs) that have the same basic level near 10^−5^ rad/s/$\sqrt{\text{Hz}}$. On the other hand, MET sensors have additional advantages in size, power consumption and price. These facts set an attractive background for further comprehensive study of MET sensors and their applications in a wide variety of areas.

The current work presents research results on the self-noise characteristics of MET sensors, which are one of the key characteristics, in a wide frequency range. At the present moment there is no unified theory of molecular electronic sensor noise, which is conditioned by the complexity and variety of the physical mechanisms responsible for MET noise. On the one hand, accidental changes of free carrier speed, caused by the irregular patterns of their dispersal, lead to fluctuations in their local density, which results in accidental diffusion currents [12]. In liquid environments, diffusion noise is imposed upon natural convection, which is also related to the concentration distribution discontinuity [13]. On the other hand, MET noise may be produced by hydrodynamic fluctuations of the liquid flow through the conversion cell [14]. Besides, random events connected with the charge transfer at the electrode-electrolyte solution interface, also contribute to the total system noise [15]. Finally, the noise generated by the electronics which preset the transducer operating voltage and provide transformation of currents in molecular electronic cells into output voltages must be taken into account [16].

In terms of the noise parameters of angular MET sensors, there is a number of publications with experimental data for several types of sensors and for various frequency ranges [17,18,19]. In particular, in [19], the distinction of noises for different types of sensors was connected with the distinction of their hydrodynamic resistance. This mechanism is apparently not the only one, which was especially proven in [19]. Unlike the mentioned works, in this study we made experimental measurements and generalized the model for a wider frequency range—from 0.01 to 200 Hz—which is, in fact, the complete frequency range of the modern applications of molecular electronic angular motion sensors. New experimental results and the corresponding theoretical explanation were found for the low-frequency portion of the spectrum. Compared to [20], direct experimental measurements of the MET sensor electrical impedance in a wide frequency range allowed us to describe the MET self-noise frequency behavior more accurately and also verifies the model better by exclusion of the frequency independent electrical resistance of a MET sensor as a fitting parameter of the model. As a result, this paper represents the first time that three dominant mechanisms of self-noise of a molecular-electronics sensor were integrated into one total theoretical model, which was confirmed with the appropriate experimental study in the whole frequency band. Based on the experimental data, the frequency range was divided into particular areas, each of which has its own predominant mechanism of self-noise generation. The purpose of the research was thus the development of a common noise model, which can be used for the complete indicated frequency range.

## 2. Experimental Section

### 2.1. Design of an Angular Motion Sensor Based on MET Technology

**Figure 1:**
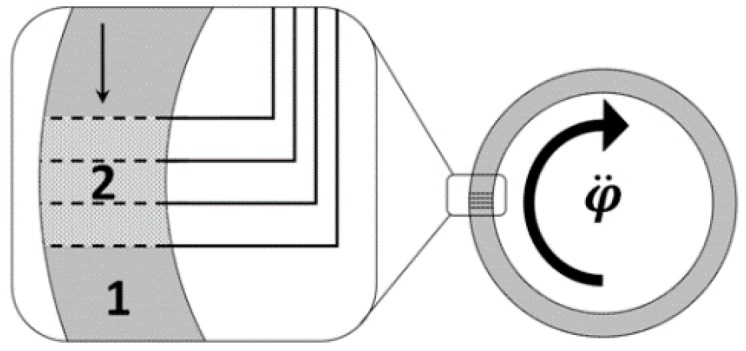
Molecular electronic angular motion sensor.1—electrolyte; 2—conversion cell.

In terms of the mechanical system, a molecular electronic angular motion sensor is not a mechanical oscillator since it does not contain a resilient returning element. An example of such a transducer is presented in Figure 1. 

The sensor is constituted by a hollow toroidal channel made of dielectric material completely filled with an air bubble-free electrolyte solution (for example, water solution of LiI with a small amount of molecular iodine I_2_). There is a conversion electrode assembly inside the torus ring, which consists of four mesh electrodes divided by dielectric perforated baffle plates, so that the liquid inside the torus ring can flow freely through the assembly. A small voltage difference (<0.9 V) is applied to the electrodes in a way that the two external electrodes have positive potential (they act as anodes) in relation to the two internal electrodes of the assembly (which act as cathodes). The electrolyte solution in the toroidal channel is both the conversion media of the mechanical signal into an electrical current and the sensor inertial mass. Under the external angular acceleration, the inertial mass starts moving relative to the transducer walls and electrodes, whilst a convection flow is created in the channel, which results in variations of the current through the converter electrodes. The current at the electrodes is sent to the concurrent electronic circuit, where it is transformed into voltage and it undergoes frequency correction and filtration. To reach the necessary MET output parameters, it is exceptionally vital to make the output signal in a wide frequency range proportional either to the angular acceleration or to the angular velocity of the object.

### 2.2. Self-Noise of Angular Motion Sensor at Low Frequencies

In accordance with the physical transduction of the mechanical effect into the liquid flow through the transductive element, molecular electronic angular motion sensors are accelerometers. All the above considered, the equation of liquid pressure in the channel of the studied angular accelerometer can be presented as follows:

(1) $$\dot{\text{q}}+\frac{{\text{SR}}_{\text{h}}}{2\text{πRρ}}\text{q}=\text{εSR}$$

where $R_{h}$ is the system hydrodynamic resistance, *S* is the channel cross-section area, *R* is the toroidal channel radius, *ρ* is the electrolyte density, $\varepsilon =\ddot{\text{φ}}$ is the angular acceleration of the accelerometer body under external effects, q is the liquid flow.

After Fourier transformation of Equation (1), we can easily define the transfer function of the mechanical system (transforming external mechanical movement (angular acceleration) into the liquid flow through the transducer) of the studied sensor:

(2) $${\text{W}}_{\text{mech}}\left(\text{ω}\right)=\frac{\text{q}}{\text{ε}}=\frac{2{\text{πR}}^{2}\text{ρ}/{\text{R}}_{\text{h}}}{1+\text{iω}/{\text{ω}}_{\text{h}}}$$

where $\omega_{\text{h}}=R_{h}S/2\pi R\rho$ is the hydrodynamic frequency, above which transfer function of the mechanical system reduces proportionally to ω^−1^ [21]. In realistic parameters the hydrodynamic resistance defining the value of ${\text{ω}}_{\text{h}}$ for this system is so high that ${\text{W}}_{\text{mech}}\left(\text{ω}\right)$ transfer function decline begins at the frequency range of tens and even hundreds of Hz. That is why for lower frequencies mechanical transfer function will be considered a constant.

Now lets’ analyze self-noise. For the studied frequency range, the self-noise of free-design molecular electronic sensor, in accordance with the formulation in [22], is defined by thermal hydrodynamic noise and geometric noise. Consequently, the mathematical expression for self-noise of a molecular electronic angular motion sensor appears as follows:

(3) $${‹{\text{ε}}^{2}›}_{\text{ω}}=\frac{{\text{R}}_{\text{h}}\text{kT}}{2{\text{π}}^{2}{\text{ρ}}^{2}{\text{R}}^{4}}+\frac{4\text{kT}}{{\text{R}}_{\text{h}}{\left|{\text{W}}_{\text{mech}}\right|}^{2}}\text{α}$$

In addition to the previous expressions, there are the following terms:

${‹{\text{ε}}^{2}›}_{\text{ω}}$ is the power spectral density of angular movement measurer self-noise in units of input angular acceleration, k is the Boltzmann’s constant, T is the absolute temperature, α is the conversion efficiency variation between the transductive element channels [22].

For the foregoing peculiarities of the conversion function of the mechanical effect into the electric current of the studied angular motion sensor and the Equation (3), it follows that at low frequencies the spectral noise density of molecular electronic angular motion sensor must be flat in units of applied angular acceleration.

### 2.3. Self-Noise of Angular Motion Sensor at Medium and High Frequencies

Studies on angular motion sensor self-noise at medium and high frequencies were performed in [20]. One of the main results of that work was the proof that the noise of the operational amplifier of the concurring electronic network prevails upon other kinds of noise at frequencies of above several tens (50–60) Hz, under the condition that the sensor impedance selection is equal to 40 Ohms. In [20], the indicated value was defined by the condition of the best correspondence to the experimental data. According to [20], electronic self-noise is mainly generated at an input stage of the electronics, where it is the result of the voltage noise of the operational amplifier input. After that simplification, the expression for power spectral density of electronic self-noise in units of applied angular acceleration can be defined as follows:

(4) $${‹{\text{ε}}_{\text{ω}}^{2}›}_{\text{electronics}}={\left(\text{ω}\right)}^{2}\cdot {‹{\text{U}}_{\text{ω}}^{2}›}_{\text{amp}}{\left|1+{\text{R}}_{\text{fb}}/{\text{Z}}_{\text{out}}\left(\text{ω}\right)\right|}^{2}\frac{1}{{\left|\text{W}\left(\text{ω}\right)\right|}^{2}}$$

Herein ω is the circular frequency, ${‹{\text{U}}_{\text{ω}}^{2}›}_{amp}$ is the noise power spectral density for input stage amplifier, $Z_{out}\left(\text{ω}\right)$ is the transducer output impedance, $R_{fb}$ is the feedback resistance of input stage amplifier, W(ω) is the angular motion sensor conversion coefficient (V/rad/s^2^).

**Figure 2:**
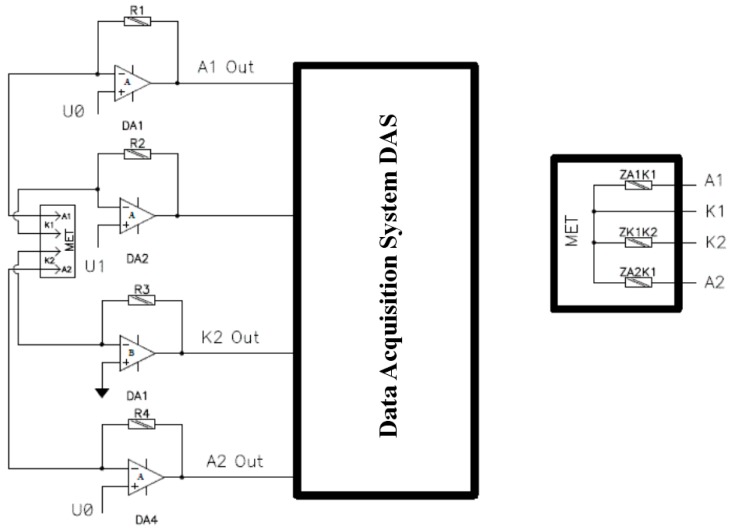
Installation diagram and equivalent electric MET scheme (insertion) for the measurement of molecular electronic angular motion transducer impedance.

The direct measurement of impedance was performed under the diagram presented in Figure 2. A molecular electronic transducer, as discussed earlier, presents four reticulate electrodes made of platinum net and installed perpendicularly to the body axis with a certain gap which is supplied by the perforated baffle plates. To set the molecular electronic cell direct current mode, a constant bias was applied from the bias source U0 (about 300 mV) to the coupled anodes (A1, A2 in Figure 2). The frequency characteristics of the transducer impedance were studied by applying bias voltage amplitude from the generator with comparatively little (≈10% of bias source U0) alternating voltage U1 to the one of the cathodes (K1 in Figure 2). To generate the alternating voltage at the studied frequencies, a FG-7002C generator was used. The current through over three electrodes was discarded from the molecular electronic cell and applied at low-noise current-to-voltage converters DA1-4, where input resistance is essentially lower than of the apparent sensors dynamic resistance. To collect the data, high resolution (24 bit) sigma-delta AD converter was used (Data Acquisition System, or DAS). To calculate the impedance, the values of the corresponding frequencies first harmonics at signal spectrum were used. According to the insertion shown in Figure 2, the whole output impedance $Z_{out}\left(\text{ω}\right)$ of MET consists of three parallel connected impedances between A1-K1, A2-K1, K1-K2, see equation below:

$$\frac{1}{Z_{out}}=\frac{1}{Z_{A1K1}}+\frac{1}{Z_{A2K1}}+\frac{1}{Z_{K1K2}}$$

Such a measurement scheme simulates the sensor equivalent output impedance and its influence on the electronic noise generated by the first stage operational amplifier according to Equation (4). In [13,23], it was experimentally shown that in the medium frequency range (from ones to tens of Hz) for the molecular electronic sensor without integral liquid flow (encapsulated sensor) through the converter, spectral dependence of noise on frequency is as follows:

(5) $${‹{\text{U}}_{\text{ω}}^{2}›}_{convective}\sim {\left(A/\surd \text{ω}\right)}^{2}$$

This kind of noise is conditioned by the occurrence of convective flows inside the transducer.

### 2.4. Experimental Results

**Figure 3:**
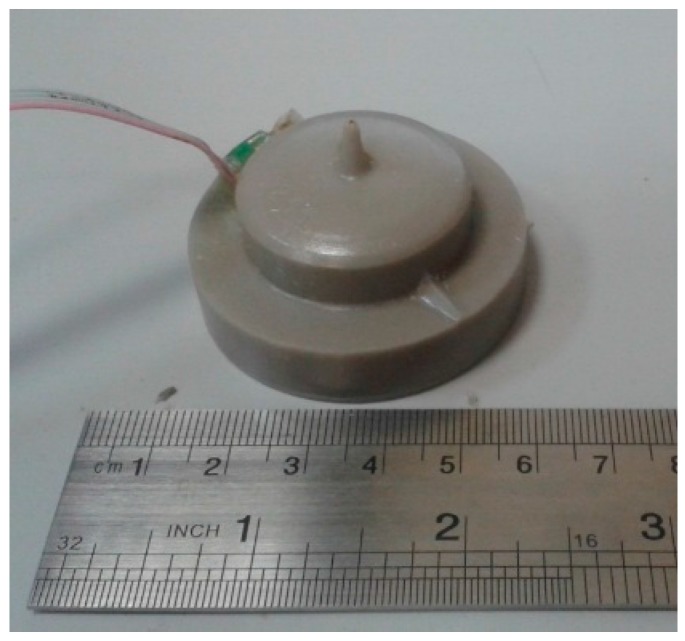
Appearance of a molecular electronic angular motion transducer.

To verify the abovementioned sensor noise models in a wide frequency range (from 0.01 to hundreds of Hz), the following experiment was performed. The experimental study of the molecular electronic angular motion transducers self-noise was performed with the sample possessing the characteristics listed in Table 1. The appearance of the studied sensor is presented in Figure 3.

**Table 1:** Structural characteristics of the studied angular motion sensors.

Characteristic	Measuring Unit	MET
Overall dimensions	mm	Ø50 × 28
Mass	grammes	less than 50
Electrolyte solution components concentration	mol/L	4 for LiJ; 0.1 for J_2_
Hydrodynamic resistance	N·s/m^5^	~10^8^
Output signal	rad/s^2^	In accordance with Figure 6
Investigated frequency range	Hz	0.01–200

**Figure 4:**
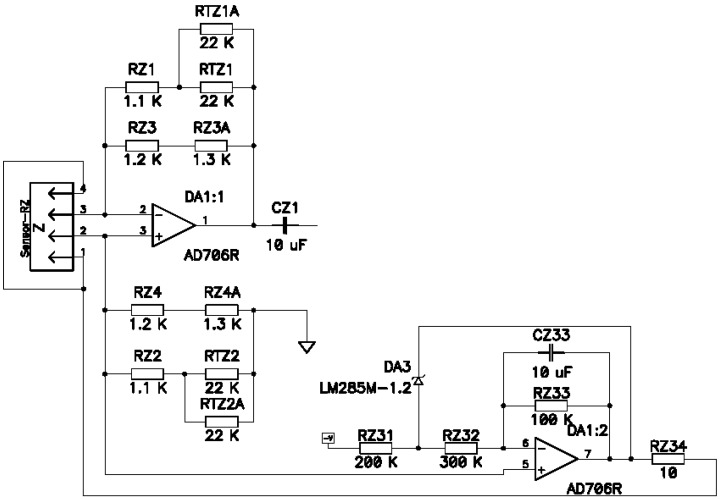
Diagram for electronic conversion unit of signal current into electric voltage.

The electronic cascade used to convert sensor signal current into voltage consisted of frequency independent amplification on an AD 706 operational amplifier (Analog Devices, Norwood, MA, USA), see Figure 4.

**Figure 5:**
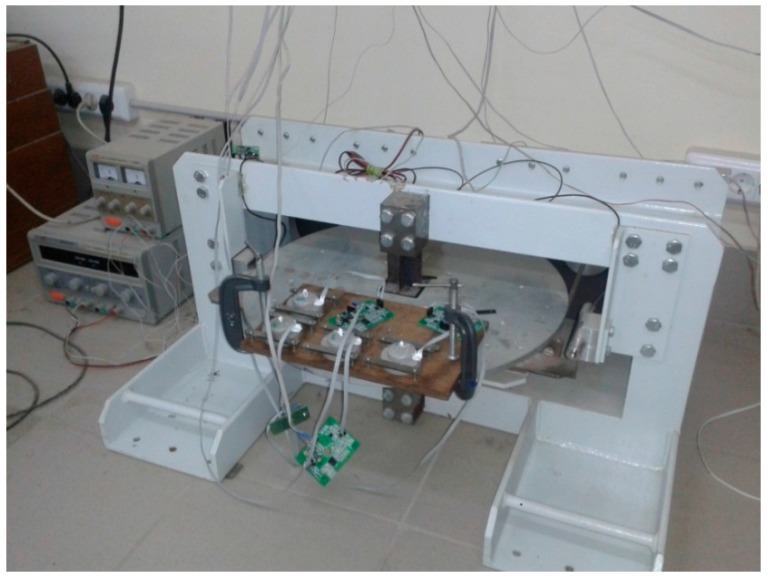
Rotatory calibration vibration platform.

The direct measurement of amplitude-frequency curve was performed on a rotatory calibrating vibration platform in the frequency range of 0.01–200 Hz (Figure 5). It was not possible to reach a wider frequency range, because of the substantial inclination impact at lower frequencies and because of the rapid fall in sensor sensitivity and lack of resolution of the vibration plate AD converter at higher frequencies.

**Figure 6:**
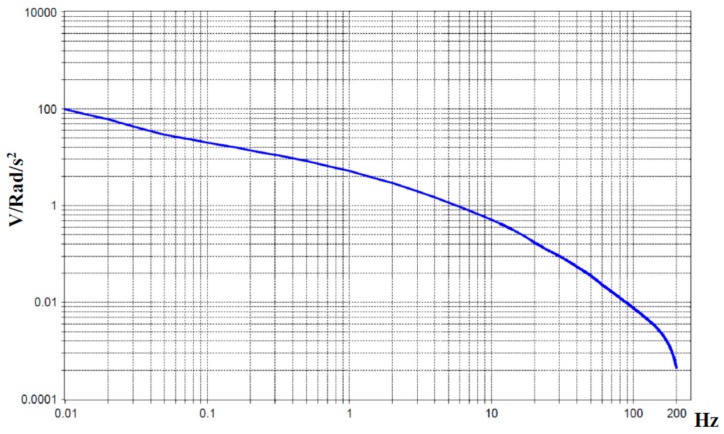
Experimental sensor conversion coefficient without frequency correction in units of angular acceleration. The Y-axis is the scale factor (V/Rad/s^2^), the X-axis is the frequency in Hz.

Figure 6 presents the measured amplitude-frequency curve of the angular sensor. It is worth noting that Figure 6 shows the amplitude-frequency curve directly for a MET transducer, which is the main sensitive element of an angular motion sensor. A molecular electronic angular velocity sensor or angular acceleration sensor will have a flat transfer function (scale factor) in a predetermined frequency range (0.01–200 Hz) proportional to the angular velocity or angular acceleration, by means of analog frequency correction with the help of a corresponding electronic board. Normally, the electronic circuit comprises several cascades of amplification and filtering for the predetermined frequency band and some thermal compensation chains [20].

Measurement of molecular electronic angular motion sensor self-noise was performed in a special basement area with a reduced amount of vibration noise. The sensor was mounted on a special concrete tabletop in a way that the sensitive axis was parallel to the gravity vector. In this orientation, the sensor detects only ground rotations relative to the vertical axis, which are typically smaller than rotations relative to horizontal axes. In turn, the reduced level of the seismic signals makes self-noise measurements more accurate. Note that according to the data presented in [23], the self-noise in a MET cell depends on its orientation relative to the gravity vector as a result of liquid mass motion under the buoyancy force, initiated by local specific density fluctuations of the electrolyte. The effect is observable in the 0.01 to 0.1 Hz range only in encapsulated cells, while in a practical rotational sensor it is masked by the hydrodynamic fluctuations represented by Equation (3).

**Figure 7:**
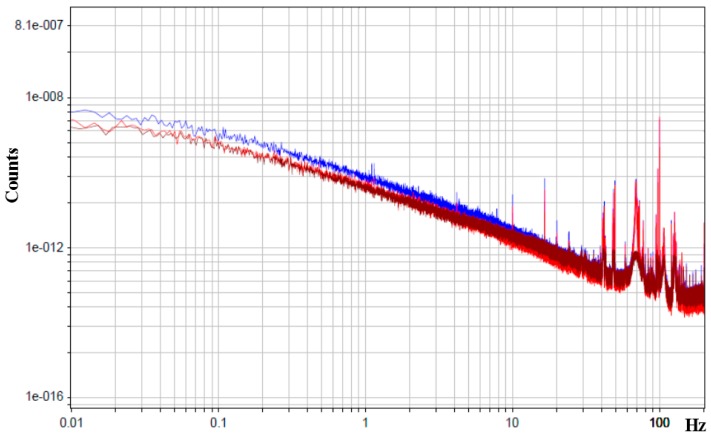
Power spectral density. The X-axis is the frequency in Hz, the Y-axis is the PSD counts. The blue and light red curves are the sensors PSD, the dark red curve is the PSD of uncorrelated part of the sensors signals.

The electronic network connected to the sensor was supplying the sensor and performing the signal current conversion amplification stage in accordance with Figure 3. To reduce the possible low-frequency background current temperature drift, the construction was covered with a foam cap. The “Out” sensor signal was sent to a 32-bit data collection system. The signal (self-noise) recording was performed simultaneously by two identical angular motion sensors, situated in close proximity to each other with a discretization frequency of 500 Hz. It should be noticed that the AD converter capacity was deliberately chosen as high, which allowed us to use only one amplification stage at high frequencies when studying sensor self-noise. In this case, the AD converter self-noise was at least one order less in comparison to the studied signal. The lack of artificial frequency compensation of high frequencies, as well as of additional noise source in the form of subsequent amplification stages substantially facilitated the study objectives of specifically defining the nature of angular motion sensor self-noise. The indicated experimental design allowed us to find the common signal component of two identical sensors on further signal processing and exclude it by calculating the correlatoring in accordance with the equations from [24]. A piece with the lowest background signal was chosen from the recorded signals (which was usually between 4 a.m. and 6 a.m.) and the power spectral density of each of the signals and of their uncorrelated part (which is, broadly defined, the molecular electronic angular motion sensor self-noise) was calculated with the Digital Signal Processing (DADiSP 6.5) software, see Figure 7. The correlation calculation of the two identical devices allowed us to reduce the “signal” component in the sensor values significantly, leaving only the device self-noise.

**Figure 8:**
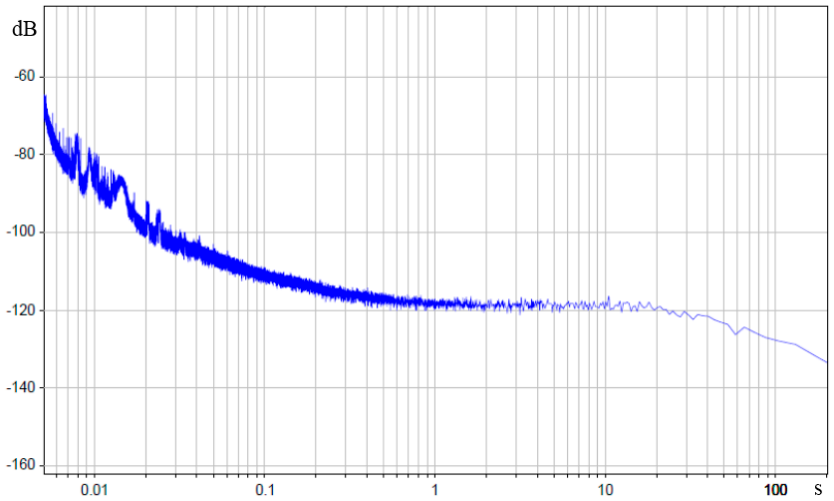
Power spectral density of molecular electronic sensor in units of input angular acceleration in decibels relating to 1 rad/s^2^/$\sqrt{Hz}$, The X-axis is the period in seconds.

Lets’ change angular motion sensor self-noise into units of input angular acceleration. To do so, we divide obtained values of sensor noise power spectral density by sensor transfer function experimental value. The result is presented in decibels relating to level in 1 rad/s^2^/√Hz in Figure 8, (0 dB = 1 $\frac{rad}{s^{2}\sqrt{Hz}}$).

Now lets’ compare the above-described theoretical models with the obtained experimental data for the specified frequency range (0.01–200 Hz, which corresponds with the periods of 0.005–100 s).

In accordance with the requirements of Equation (3), the results of the experimental noise measurements of Figure 8 were transformed into units of input angular acceleration.

It can be seen that the resulting power spectral density has practically no frequency dependence in the range of 0.025–2 Hz (0.5–40 s) and is flat in units of applied acceleration, which totally agrees with the Equations (2) and (3).

Therefore, we can draw a conclusion that to describe angular motion sensor self-noise in the low frequencies range (0.025–2 Hz) in units of applied angular acceleration it is enough to take account of hydrodynamic thermal noise, which agrees with the following simplification of the Equation (3):

(6) $${‹{\text{ε}}_{\text{ω}}^{2}›}_{Rh}=\frac{{\text{R}}_{\text{h}}\text{kT}}{2{\text{π}}^{2}{\text{ρ}}^{2}{\text{R}}^{4}}$$

The observed decline in noise pattern in the lower frequency range can be described by low quality measurement of the sensor transmission characteristic on a vibration plate, as microscopic inclinations of the vibration plate contribute to the measurement errors at the lowest frequencies, which, in its turn, leads to the increased values of characteristic transmission.

**Figure 9:**
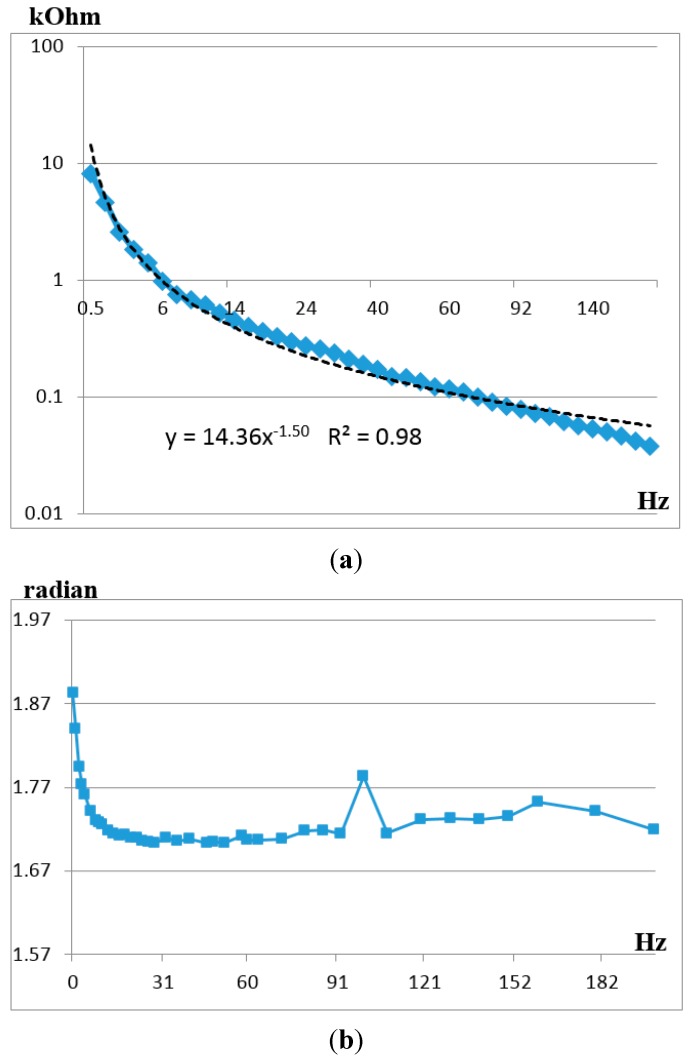
(**a**) Experimental impedance module of molecular electronic angular motion sensor in kilohms; (**b**) Experimental impedance phase of molecular electronic angular motion sensor in radians. The X-axis is the frequency in Hz.

According to the method of direct measurement of molecular electronic angular motion sensor electrical impedance, which was described above for the range of medium and high frequencies, the experimental observation of range and phase of the electrical impedance was performed. The results are presented in Figure 9a, which shows the impedance module and the corresponding approximation line, and in Figure 9b, which shows the impedance phase. It is important to note that the impedance module in the studied frequency range approaches the curve ~1/ω^3/2^ most accurately, whereas the phase shift is close to π/2. The sensor impedance is defined by the classical capacity of the double electrical layer at electrode-electrolyte interface, which has been studied in a large number of both fundamental theoretical and experimental works [25,26,27,28,29].

The modelling of the contribution to sensor self-noise of the operational amplifier of conversion stage of signal current into voltage with molecular electronic sensor with complex impedance value in input from Figure 9 was performed in accordance with Equation (4).

**Figure 10:**
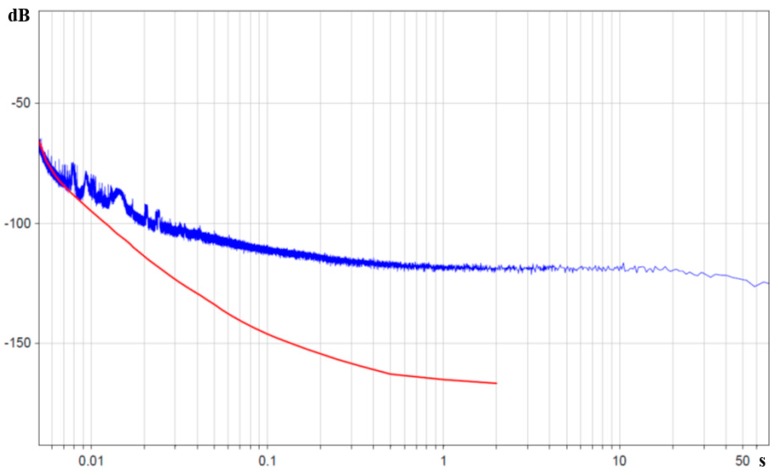
Power spectral density of molecular electronic sensor noise in units of input angular acceleration in decibels relative to 1 rad/s^2^/$\sqrt{\text{Hz}}$ is the blue curve, whereas noise of operational amplifier AD706 with input sensor impedance is the red curve. The X-axis is the periods in seconds.

Data from the AD706 microchip manufacturer [30] give a value of ${‹U_{\text{ω}}^{2}›}_{amp}^{1/2}\sim 20\frac{\text{nV}}{\sqrt{\text{Hz}}}$ with the ascent up to $40\frac{\text{nV}}{\sqrt{\text{Hz}}}$ to lower frequencies (0.5 Hz). By further setting the measured values of $Z_{in}$, $R_{fb}$= 462 Oh and by expressing the received dependence in decibels relative to 1 rad/s^2^/$\sqrt{\text{Hz}}$, we get the red curve in Figure 10.

The obtained experimental data substantially improve the accuracy of the assumptions in [20] by using the real values of angular motion transducer impedance, as well as considering impedance frequency dependence. In accordance with Figure 10 the electronic noise starts prevailing over converter noises of other kinds only at frequencies higher than 90–100 Hz.

In accordance with the above-described convective noise model, for the medium frequency range of 2–100 Hz, the noise with power spectral density inversely proportional to frequency must be observed, according to Equation (5). Modelling of the frequency dependence by experimental points in the stated range gives the following equation:

(7) $${‹{\text{U}}_{\text{ω}}^{2}›}_{\text{ convective}}^{1/2}=0.0000041\cdot {\text{ω}}^{-0.55}$$

In the units of applied angular acceleration for power spectral density:

(8) $${‹{\text{ε}}_{\text{ω}}^{2}›}_{\text{ convective}}=1.68\cdot 10^{-11}\cdot \omega^{-1.1}\cdot \frac{1}{{\left|W\left(\omega \right)\right|}^{2}}$$

Herein W(ω) is the angular motion sensor conversion coefficient (V/rad/s^2^).

**Figure 11:**
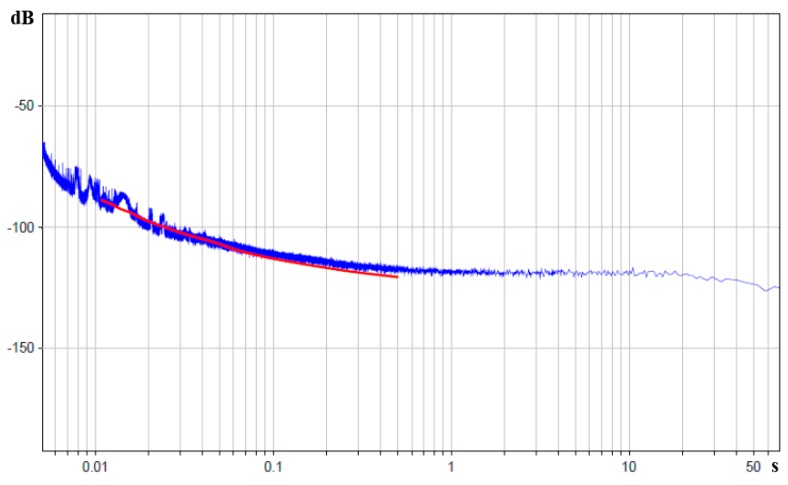
Power spectral density of molecular electronic sensor noise in units of input angular acceleration in decibels relative to 1 rad/s^2^/$\sqrt{\text{Hz}}$ is the blue curve, theoretical curve corresponding to dependence Equation (7) is the red curve. The X-axis is the periods in seconds.

Root-mean-square deviation of the experimental data from the fitted dependence Equation (8) is not higher than 17% in the range of 2–100 Hz. Figure 11 shows the comparison of the experimental part of noise curve with the fitted dependence Equation (8) in decibels relating to 1 rad/s^2^/$\sqrt{\text{Hz}}$.

The resulting agreement of the experimental data with the results of previous works [13,23] implies that in the frequency range of 2–100 Hz, molecular electronic angular motion sensor self-noise has a convective nature and corresponds to the earlier described noise in encapsulated molecular electronic transducers, where the possibility of integral liquid flow through the transductive element is excluded.

After root-mean-square addition of the observed noise mechanisms, we get the general Equation (9):

(9) $${‹{\text{ε}}_{\text{ω}}^{2}›}_{total}={‹{\text{ε}}_{\text{ω}}^{2}›}_{Rh}+{‹{\text{ε}}_{\text{ω}}^{2}›}_{convective}+{‹{\text{ε}}_{\text{ω}}^{2}›}_{electronics}$$

**Figure 12:**
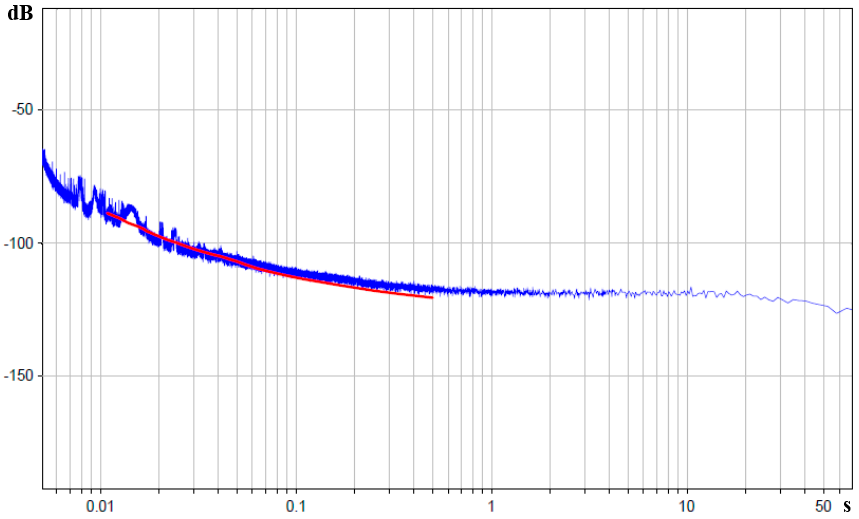
Power spectral density of molecular electronic sensor noise in units of input angular acceleration in decibels relative to 1 rad/s^2^/$\sqrt{\text{Hz}}$ is the blue curve, theoretical curve corresponding to root-mean-square sum of three noise mechanisms is the red curve. The X-axis is the periods in seconds.

On performing such addition of the theoretical noise models of different kinds and comparing them to the experimental data, we get Figure 12.

## 3. Conclusions

The results presented in this work show that three main noise process mechanisms prevail in molecular electronic angular motion sensors in the frequency range between 0.01 and 200 Hz. For low frequencies of 0.01–2 Hz, it is the noise related with hydrodynamic thermal fluctuations, which is a kind of blank noise in units of applied angular acceleration and is described by the Equation (6).

In the medium range of frequencies of 2–100 Hz, it is the noise described in [13,23] as convection noise with the dependence of spectral density on frequency in accordance with the Equation (8).

At high frequencies of 100–200 Hz, the model from [20] was specified by direct measurement of impedance and it was shown that the noise in the stated frequency range corresponds to the operational amplifier noise with the load of molecular electronic angular motion sensor impedance, and is described by the Equation (4).

The observed agreement of the experimental behavior of self-noise in the studied frequency range and Equation (9) supports the above-discussed theoretical model.
